# Profile of Patients With Tuberculous Pleural Effusion in Qatar: A Retrospective Study

**DOI:** 10.7759/cureus.12391

**Published:** 2020-12-31

**Authors:** Muhammad Zahid, Vamanjore A Naushad, Nishan K Purayil, Muhammad B Jamshaid, Jessiya Parambil, Farah Rashid, Shanima Ismail, Muhammad Saddique, Sajid Chalihadan

**Affiliations:** 1 Medicine, Hamad Medical Corporation, Doha, QAT; 2 Internal Medicine, Hamad General Hospital/Hamad Medical Corporation, Doha, QAT; 3 Internal Medicine, Weill Cornell Medical College, Doha, QAT; 4 Internal Medicine, College of Medicine, Qatar University, Doha, QAT; 5 Internal Medicine, Hamad Medical Corporation, Doha, QAT

**Keywords:** tuberculosis, pleural effusion, bmi, lights criteria, tuberculous pleural effusion, acid-fast bacillus

## Abstract

Introduction

Tuberculosis (TB) remains one of the top 10 causes of death globally. Around 1.7 billion people are infected with mycobacterium TB worldwide, and almost 90% of cases each year are found in 30 high TB burden countries. Due to the influx of a large expatriate population mainly from the high TB burden countries, there is an increased number of pulmonary TB as well as tuberculous pleural effusion cases reported in Qatar.

Objectives

The demographic, clinical, laboratory, and histopathological parameters of patients with tuberculous pleural effusion were assessed.

Methods

A single-center study was conducted at Hamad General Hospital, Hamad Medical Corporation, Doha, Qatar. Adults diagnosed to have tuberculous pleural effusion were included, and those with clinical suspicion of tuberculous pleural effusion with positive sputum acid-fast bacillus (AFB) but negative AFB in pleural samples were excluded.

Results

A total of 106 patients were reviewed, of whom 100 were included for the final analysis, with 86% being men. Majority were from the Asian subcontinent, and the mean age was 33.8 years (SD ± 10.3). Main symptoms in decreasing order were cough (77%), fever (56%), and chest pain (54%). Of the patients, 72% had normal BMI, and rest were above the normal range. Anemia and hypoalbuminemia were found in 36.7% and 89.8% of the patients, respectively. Positive AFB culture was observed in pleural biopsy (79%), pleural fluid (13%), and sputum (16%). Positive AFB by polymerase chain reaction (PCR) was observed in pleural biopsy (57%), pleural fluid (3%), and sputum (2.2%), whereas AFB smear was positive in 2% of pleural biopsy samples. Caseating granuloma was seen in 80% of patients. All the three Light's criteria were met by 30% of the patients whereas 52% had two criteria fulfilled. No association between the number of Light's criteria and AFB yield was observed.

Conclusions

TPE was more common in healthy young adults. The AFB yield on pleural biopsy, PCR, and culture was significantly higher than that on all other samples. The number of positive Light's criteria did not have any association with positive AFB yield.

## Introduction

Tuberculosis (TB) remains one of the top 10 causes of death globally. According to the World Health Organization (WHO) 2019 report, around 1.7 billion people are infected with mycobacterium TB (MTB) globally, and almost 90% of cases each year are found in 30 high TB burden countries. The case rates vary from country to country, ranging from less than 50/million population/year to more than 5000/million population/year [1]. In 2018, there were 1.2 million deaths due to TB in human immunodeficiency virus (HIV) negative people. According to the WHO 2019 report, most cases are found in Southeast Asia (44%), Africa (24%), and the Western Pacific region (18%). Eight countries accounted for two-thirds of total global cases of TB, that is, India (27%), China (9%), Indonesia (8%), Philippines (6%), Pakistan (6%), Nigeria (4%), Bangladesh (4%), and South Africa (3%) [2].

Extrapulmonary TB is not an uncommon entity, especially in areas where TB is endemic. Tuberculous pleural effusion (TPE) is one of the most common extrapulmonary TB after TB lymph node. One study carried out in the eastern part of Kingdom of Saudi Arabia reported that 37% of pleural effusion were due to TB [3], whereas a study conducted in Qatar on 200 pleural effusion patients found that the most common cause of effusion was TB (32.5%), and majority of them were from the Asian subcontinent [4]. The incidence of pleural involvement varies from 3-5% in TB non-endemic areas to 30% in TB endemic areas [5,6].

The accumulation of fluid in TPE is multifactorial. The MTB antigen enters the pleural space following a rupture of subpleural caseous focus, which causes inflammatory response and results in increased capillary permeability. This also obstructs the lymphatic outflow, resulting in decreased clearance of pleural fluid [7-9]. TPE most commonly presents with fever (85%), cough (70%), and pleuritic chest pain (75%). Dyspnea (50%), night sweats (50%), and weight loss (25%) can also be there. TPE is commonly unilateral and the size of the effusion varies from patient to patient [10].

Even though Qatar is not listed in the high TB burden countries, a high number of pulmonary TB as well as TPE cases are reported due to the influx of a large expatriate population mainly from the high TB burden countries. In this single-center study, we sought to know the epidemiological, clinical, and biochemical profile of patients who were diagnosed with TPE.

## Materials and methods

Study design and setting

A retrospective observational study was carried out at the Hamad General Hospital, Hamad Medical Corporation, Doha, Qatar.

Study population

Patients older than 18 years of age who were admitted to the medical ward and diagnosed to have TPE between the period of January 2016 to December 2019 were included. The diagnosis of TPE was based on any of the following criteria: (1) positive acid-fast bacillus (AFB) by polymerase chain reaction (PCR)/smear or culture in the pleural fluid, (2) positive AFB by PCR/smear or culture in the pleural biopsy samples, and (3) caseating granuloma seen in pleural biopsy samples. Patients with clinical suspicion of TPE with positive sputum AFB but negative AFB in pleural samples were excluded.

Study procedures

Patient data were retrieved from the clinical information system using the health care number of study population. This included demographic features, body mass index (BMI), comorbid conditions, clinical symptom and signs, radiological data (chest X-ray, CT scan of the chest), blood investigations, pleural fluid analysis, and thoracoscopy /histopathology findings.

Ethical consideration

The study protocol was approved by the Ethics Committee of Medical Research Center of the institution.

Statistical analysis

Descriptive statistics were used to summarize and determine the sample characteristics and distribution of various considered parameters related to demographic and clinical features, biochemical parameters, and histopathological characteristics. The normally distributed data and results are reported with mean and standard deviation (SD). Categorical data are summarized using frequencies and percentages. Associations between two or more qualitative variables were examined and assessed using Pearson’s chi-square test and Fisher’s exact test as appropriate. Unpaired t-test and analysis of variance (ANOVA) were used to compare mean values of different quantitative parameters (such as age, BMI, white blood cell count, C-reactive protein (CRP), and other biochemical parameters) between two or more groups (such as AFB, histopathology, and treatment details). All statistical analyses were performed using statistical packages SPSS Version 24.0 (IBM Corp., Armonk, NY, USA) and Epi Info 2000 (Centers for Disease Control and Prevention, Atlanta, GA, USA).

## Results

Basic demography

A total of 106 files were reviewed for inclusion, of which 100 patients fulfilled the criteria for analysis after exclusion (Incomplete data: 3; non-TB effusion: 3). Majority (86%) of the patients were men, and the mean age was 33.85 years (range: 18-84 years; SD± 10.26). Of the study patients, 60% were from the Asian subcontinent. Cough (77%), fever (56%), and chest pain (54%) were the most common presenting symptoms. Also, 72% had normal BMI (18.6-25). None of the patients was underweight (i.e., with BMI less than 18.5). The basic demographics and clinical features are summarized in Table 1.

**Table 1:** Basic demographic characteristics and clinical profile TB, tuberculosis; COPD, chronic obstructive pulmonary disease; HIV, human immunodeficiency virus; SOB, shortness of breath; PPD, purified protein derivative; INH, isoniazid; MDR-TB, multidrug-resistant tuberculosis

Characteristics	Number (%), n = 100
Gender	
Male	86 (86)
Female	14 (14)
Age (mean ± SD), years	
Mean age	33.85 ± 10.26
<30	45 (45)
30-50	50 (50)
>50	5 (5)
Length of stay (days ± SD)	
Mean length of stay	8.34 (±3.54)
Nationality	
Qatar	1 (1)
Africa	25 (25)
India	19 (19)
Nepal	28 (28)
Sri Lanka	2 (2)
Bangladesh	9 (9)
Philippines	13 (13)
Indonesia	1 (1)
Pakistan	2 (2)
Childhood TB	
Yes	3 (3.57)
No	84 (96.3)
Missing data	13
Contact with open TB	
Yes	10 (10.3)
No	87 (89.7)
Missing data	3
Comorbidities	
Nil	89
Diabetes	6 (6)
Hypertension	2 (2)
Asthma/COPD	5 (5)
Kidney Disease	1 (1)
HIV	0
Symptoms	
Fever	56 (56)
Cough	77 (77)
Weight loss	25 (25)
Night sweats	29 (29)
Chest pain	54 (54)
SOB	34 (34)
Anorexia	15 (15)
others	17 (17)
Duration of symptoms (days)	
<7	26 (26)
8 to 15	34 (34)
16-30	28 (28)
>30	12 (12)
BMI	
<18.5	0
18.6-25	72 (72)
25.1-30	26 (26)
>30	2 (2)
PPD	
<5 mm	14 (34.1)
6-15 mm	9 (22)
>15 mm	18 (43.9)
missing data	59
QuantiFERON®	
Positive	37 (75.5)
Negative	12 (24.5)
Missing data	51
Other TB manifestation	
Parenchymal involvement	10 (10)
Lymph nodes	3 (3)
Pott’s spine	1 (1)
Pericardial	1 (1)
Peritoneal	3 (3)
Disseminated	1 (1)
Drug resistance	
No	95
INH	3
Rifampicin	2
MDR-TB	
No	98
Yes	2

Laboratory parameters

Around 36.7% of the patients had anemia. In 44.9%, the platelet count was found to be high. Majority of the patients had hypoalbuminemia (89.8%). CRP was elevated in all patients except three, and 53.9% of patients had a CRP of more than 100 (Table 2).

**Table 2:** Laboratory parameters and thoracoscopy findings CXR, chest X-ray; LDH, lactate dehydrogenase

Variable	Number (%), n = 100
Hemoglobin	
Normal	62 (63.3)
Anemia	36 (36.7)
Missing	2
White blood cell count (10^3^/uL)	
4,000-10,000	89 (89.9)
<4,000	0
>10,000	10 (10.1)
Missing data	1
Lymphocytes	
Normal	59 (60.2)
Lymphopenia	39 (39.8)
Lymphocytosis	0
Missing data	2
Platelets (10^3^/uL)	
150-400	52 (53.1)
>400	44 (44.9)
<150	2 (2)
Missing data	2
C-reactive protein (mg/L)	
0-5	3 (3.4)
6-100	38 (42.7)
101-200	47 (52.8)
>200	1 (1.1)
Missing data	11
Albumin (gm/L)	
35-52	10 (10.2)
<35	88 (89.8)
Missing data	2
CXR effusion	
Right sided	52 (52)
Left sided	46 (46)
Bilateral	2 (2)
CXR: parenchymal involvement	
Yes	10 (10)
Pleural fluid: lymphocytes	
>50%	87 (88.8)
<50%	11 (11.2)
Missing data	2
Light’s criteria	
Protein ratio > 0.5	97 (97)
LDH ratio > 0.6	52 (52)
LDH > two-thirds of serum LDH	63 (63)
Number of Light’s criteria fulfilled	
1	18 (18)
2	52 (52)
3	30 (30)
Therapeutic thoracocentesis	19 (19)
Pleural biopsy: histopathology	
Caseating/necrotizing granuloma	80 (80)
Noncaseating granuloma	13 (13)
Others	7 (7)
Thoracoscopic findings	
Granuloma	11 (11.57)
Adhesions	42 (44.21)
Inflamed pleura	77 (81.05)
Sago-like nodules/nodules	52 (4.74)
Incomplete data	5
Complication of thoracoscopy	
Nil	89 (89)
Pneumothorax	9 (9)
Hydropneumothorax	2 (2)

Radiological findings

Of the study patients, 52% had right-sided pleural effusion and 46% had left-sided effusion on their chest X-rays. Only two patients had bilateral pleural effusion on their chest X-ray. Parenchymal involvement in chest X-ray was noted in 10% of the patients (Table 2).

Pleural fluid analysis

Overall, 87% of the patients had lymphocyte predominance (>50% lymphocytes) in the pleural fluid, and almost all except three had a pleural fluid/serum protein ratio of more than 0.5. On analyzing patients fulfilling the Light's criteria, all three criteria were met by 30% of the patients, whereas 52% had two criteria fulfilled. The details of pleural fluid analysis are summarized in Table 2.

Thoracoscopy and pleural biopsy findings

All patients underwent thoracoscopy. Inflamed pleura (81%) and nodules (54.7%) were the most common findings observed in thoracoscopy. On histopathological examination of the biopsy sample, necrotizing/caseating granuloma was seen in 80% of the study patients. Eleven patients developed complications after thoracoscopy (pneumothorax in nine and hydropneumothorax in two) (Table 2).

AFB yield by different specimens and different methods

The yield of AFB by smear was low in all three samples (sputum: 0; pleural fluid: 0; and pleural biopsy: 2%). Even though the positivity of AFB by PCR technique in sputum and pleural fluid was low (2 and 3, respectively), it was significantly high in biopsy samples (57%). Similarly, AFB culture in biopsy specimen showed significantly higher positivity (79%) than sputum or pleural fluid culture (15% and 13%, respectively) (Table 3).

**Table 3:** AFB yield from various sources PCR, polymerase chain reaction: AFB, acid-fact bacillus

Variable	AFB yield		
Sputum	Positive	Negative	Missing data
Smear	0	92	8
PCR	2	87	11
Culture	15	78	7
Pleural fluid			
Smear	0	100	
PCR	3	97	
Culture	13	87	
Pleural biopsy			
Smear	2	98	
PCR	57	43	
Culture	79	21	

Sputum AFB positivity with normal chest X-ray

In patients with no obvious parenchymal involvement on chest X-ray, sputum for AFB was positive by PCR and culture in 2.2% (2/90) and 11.1% (10/90), respectively.

Comparison of parameters based on Light's criteria

We divided the patients into three groups based on the number of fulfilled Light's criteria and compared the laboratory parameters, mainly the positive yield of AFB, using different methods. There was no significant difference in the positive yield of AFB by different techniques in samples from sputum, pleural fluid, and pleural biopsy, and the three Light's criteria groups (Table 4).

**Table 4:** Correlation of variables with Light's criteria WBC, white blood cell; CRP, C-reactive protein; PPD, purified protein derivative; AFB, acid-fast bacillus; PCR, polymerase chain reaction; INH, isoniazid

Variables	Met one criterion	Met two criteria	Met three criteria	Total	p-Value
Blood: WBC count (10^3^/uL)					
Normal (4,000-10,000)	18	43	28	89	0.041
More than 10,000	0	9	1	10	
Lymphocytes					
Normal	12	30	17	59	0.627
Lymphopenia	5	22	12	39	
CRP (mg/L)					
0-5	0	3	0	3	0.004
6-100	11	13	14	38	
101-200	3	32	12	47	
>200	1	0	0	1	
Quanti-FERON					
Negative	3	5	4	12	0.757
Positive	6	19	12	37	
Mantoux (PPD)					
<5 mm	3	5	6	14	0.932
6-15 mm	2	4	3	9	
>15 mm	5	8	5	18	
Chest X-ray: parenchymal involvement					
No	13	47	30	90	0.008
Yes	5	5	0	10	
BMI					
18.5-25	15	39	18	72	0.178
25.1-30	2	13	11	26	
>30	1	0	1	2	
Serum albumin (gm/L)					
Normal	3	6	1		0.276
Less than 35	14	46	28		
Pleural fluid: lymphocyte					
>50%	17	43	27	87	0.099
<50%	0	9	2	11	
Pleural fluid AFB: smear					
Positive	0	0	0		
Negative	18	52	30	100	
Pleural fluid: AFB PCR					
Positive	0	3	0	3	0.24
Negative	18	49	30	97	
Pleural fluid: AFB culture					
Positive	4	8	1	13	0.129
Negative	14	44	29	87	
Pleural biopsy: AFB smear					
Positive	2	0	0	2	0.01
Negative	16	52	30	98	
Pleural biopsy: AFB PCR					
Positive	12	25	20	57	0.172
Negative	6	27	10	43	
Pleural biopsy: AFB culture					
Positive	15	37	27	79	0.115
Negative	3	15	3	21	
Drug resistance					
Nil	15	50	30	95	0.003
INH	3	0	0	3	
Rifampicin	0	2	0	2	

## Discussion

In this study, we sought to know the profile of patients with TPE in a tertiary care hospital in Qatar. All the study patients except one belonged to the expat population mainly from high TB burden countries. More than half of our study cohort was from the Asian subcontinent, mainly Nepal, India, followed by the African continent. The number of native Qatari nationals and patients from the western world with TPE were negligible. From the clinical point of view, most patients were young, healthy patients with no significant prior comorbid conditions and majority presented with cough and fever.

We observed that majority of our study population had normal BMI and few were in the overweight category. Contrary to the regular belief, none of the patients was underweight. Several studies have been conducted in the past on the association between BMI and TB and have shown conflicting results. A study conducted on two groups of children, one with pulmonary TB and the other with extrapulmonary TB, showed that the proportion of overweight or obese boys was higher in the group with extrapulmonary TB (32.4%) compared to the group with pulmonary TB (13.8%) [11]. Another study conducted on patients older than 65 years reported that obese and overweight persons have a significantly lower risk of developing active TB when compared to normal-weight individuals [12]. Others studies have shown the association between low BMI with high risk of TB [13] and severity of disease [14,15].

Almost half of our study patients had elevated platelet count. Platelet count and mean platelet volume (MPV) have been studied before as inflammatory markers in TB. One study showed significantly high platelet count and MPV in patients with active TB, which decreased with anti-tuberculous treatment [16], whereas, another study carried out by Lee et al. showed significantly high MPV and platelet count in TB. Their results revealed a direct correlation between MPV and CRP in TB but no correlation between platelet count and CRP [17]. The pathophysiological mechanism by which platelets acts as inflammatory cells includes activation of complements, release of inflammatory mediators, and increased vascular permeability [18].

Our results showed that the yield of AFB smear and PCR on pleural fluid was very low and negligible (0% and 3%, respectively). The literature showed that microscopic smear with Ziehl-Neelsen (ZN) stain of pleural fluid has a yield of fewer than 10% [19], which may increase to 20% in HIV-positive people with effusion, TB empyema, and loculated effusion [20,21]. Nucleic acid amplification test (NAAT) by PCR, which detects MTB genetic material, has a sensitivity ranging from 28% to 81% for pleural fluid and 90% for pleural tissue and a specificity of 90% to 100% [22]. Similar to our observation, previous studies conducted by Antonio et al. also showed a similar result. In their study, only two out of 79 patients were positive for ZN stain of pleural fluid, and eight (13.7%) out of 52 patients were positive for PCR [23]. Low bacterial load and the presence of PCR inhibitors in pleural fluid have been shown as possible reasons for this low yield in pleural fluid [24]. The positive yield from pleural fluid AFB culture in our study was only 13%, which is in agreement with the results of Antonio et al. (16.6%) [23]. The yield of mycobacterial culture from pleural fluid varies according to the culture media, ranging from less than 30% in Lowenstein-Jensen medium culture to 70% in BACTEC-MGIT medium culture [19,25].

Similar to pleural fluid, the yield of AFB smear in pleural biopsy was also low in our study (2%), whereas yield of PCR and culture of pleural biopsy samples were high, with culture (79%) showing a significantly higher positivity rate than PCR (57%). This is in contrast to the results of a past study that reported a diagnostic accuracy of 23.5% by smear or PCR or culture of biopsy sample [23]. Histopathological results in our study showed a very high percentage of the presence of granuloma in the study samples (80%), which is in contrast to the previously reported results of 41.2% [23]. Pleural biopsy has a sensitivity of 69%-97%, which is higher in HIV-positive patients [20,22]. The technique used for obtaining biopsy also has an effect on sensitivity. Medical thoracoscopy shown to have a sensitivity of upto 100% for TB pleuritis and increases the yield for culture and PCR [26,27], whereas ultrasound-guided biopsy has a diagnostic yield of 90% [28].

We also evaluated the sputum AFB positivity in patients with no obvious parenchymal involvement in chest X-ray. It was observed that sputum sample was bacteriologically positive (PCR or culture) in 15% (15/100) of patients with TPE, out of which 10% had no parenchymal involvement on chest X-ray. This has clinical significance in deciding the treatment regimen of the patient and also has epidemiological implications. In routine clinical practice, patients with TPE without obvious parenchymal involvement on chest X-ray are considered noninfectious. However, positive AFB in sputum in such patients renders them infectious, which needs isolation as well as contact tracing. In addition, the treatment regimen of such patients varies from that of TPE with negative sputum AFB. In comparison to our observation, previous studies have reported a higher percentage of positivity in sputum. Chaudhuri et al. evaluated the role of sputum examination for AFB in TPE patients without parenchymal involvement and reported that in 22.2% of their patients the sputum AFB was positive by smear and/ or culture [29]. Conde et al. studied the yield of AFB smear and culture in induced sputum in TPE and found that bacteriological yield was 55% for sputum culture in patients with normal chest X-ray [30].

## Conclusions

In the state of Qatar, TPE was more common in young healthy adults from high TB burden countries. None of the study patients was underweight, giving the assumption that low BMI is not a vulnerability factor for TPE. The AFB yield on pleural biopsy PCR and culture was significantly higher than that on all other samples. The number of positive Light's criteria did not have any association with positive AFB yield. Finally, we recommend that sputum for AFB tests should be conducted in all patients with TPE even with no obvious parenchymal lesion on chest X-ray.
